# Abdominal compartment syndrome complicating necrotizing enterocolitis: A case report

**DOI:** 10.1016/j.amsu.2021.102961

**Published:** 2021-10-30

**Authors:** James G. Glasser

**Affiliations:** Saint Peter's University Hospital, Rutgers Medical School, New Brunswick, NJ, USA

**Keywords:** Necrotizing enterocolitis, Abdominal compartment syndrome, Septic shock, Multisystem organ failure

## Abstract

**Introduction:**

There are several disease entities subsumed under the heading Necrotizing Enterocolitis (NEC):

**1:**

The infectious enterocolitis that causes bowel necrosis.

**2:**

Spontaneous Intestinal Perforation which is linked to the use of Indocin to hasten closure of a patent ductus arteriosus (PDA); the perforation occurs in bowel that is well perfused and viable.

**3:**

Perforations that occur in bowel that is obstructed by thick or inspissated meconium (Awolaran and Sheth, Sept 2021) [1].

**4:**

The uncommon variant that is associated with the abdominal compartment syndrome.

**Case report:**

A case is presented in which a preemie suddenly developed massive abdominal distension. The neonatologist embarked upon the usual work-up and therapeutic interventions but was stymied by the inability to pass an orogastric tube to relieve the abdominal distension.

**Discussion:**

The purpose of this report is not to criticize the neonatologist, but to emphasize the difference between this case, complicated by the abdominal compartment syndrome, and the usual case of NEC.

**Conclusion:**

This is an unusual manifestation of NEC; and in my experience, it is uniformly fatal. Like many diseases with a fulminant course, our therapeutic efforts seem always too little, too late.

Perhaps, by calling attention to this unusual association, its dismal outcome may be altered.

## Highlights

• A preemie may suddenly become septic and develop abdominal distension. Is this primary enterocolitis or ileus secondary to a septic focus elsewhere?
• The abdominal radiographs may have a soap bubble appearance - generalized dilatation of the intestine and thinning of the bowel wall.
• In addition to being a sign of sepsis, abdominal distension may become a deleterious component in its pathogenesis. The abdominal compartment syndrome, by decreasing venous return and limiting diaphragmatic excursion, exacerbates cardiorespiratory failure.
• Neonatologists use familiar, safe modalities - pharmacopeia and ventilation - to prepare their fragile patients for surgery.
• By diminishing the intra-abdominal pressure, laparotomy works in tandem with these modalities. Venous return and cardiac output are improved (metabolic acidosis); and diaphragmatic excursion and ventilation are facilitated (respiratory acidosis).

## 1 Introduction

Necrotizing enterocolitis (NEC) is a progressive illness that begins with feeding intolerance: increased gastric residuals, abdominal distension, blood in the stool, and desaturation episodes. The baby's abdomen may be soft initially; but over time, signs of peritonitis develop. The hallmark of NEC is pneumatosis, on abdominal radiographs. Medical therapy is instituted, to which the disease either responds; or it progresses, ultimately requiring surgery.

Spontaneous intestinal perforation (SIP) is surprising when it occurs. The baby usually is doing well; then an astute nurse notices some subtle change, and obtains an X-ray, which demonstrates pneumoperitoneum.

The subject of this report is entirely different. The onset of the disease is sudden; its progression is rapid; and the abdominal distension is so profound as to become a component in the pathogenesis of the disease. The abdominal compartment syndrome adds urgency and complexity to the treatment of a profoundly ill infant.

Abdominal distension is limited by the compliance of the abdominal wall. Once this is exceeded, the intra-abdominal pressure rises precipitously and results in the abdominal compartment syndrome, whose injurious effect rivals host resistance and virulence of pathogen as determinative of survival.

The abdominal compartment syndrome produces hypotension and exacerbates respiratory failure. Sepsis causes multisystem organ failure. The two processes work in tandem; both must be treated expeditiously. By relieving the intra-abdominal pressure, laparotomy restores venous return and cardiac output; it facilitates diaphragmatic excursion and correction of acidosis (metabolic and respiratory) far more expeditiously than vasopressors or ventilatory adjustments, increasing PIP (peak inspiratory pressure) or PEEP (positive end expiratory pressure).

## 2 Case report - presented in accordance with SCARE guidelines [2]

A 700-g, 28 weeks AGA, boy was delivered by cesarean section. His Apgar score was 7 at 1 min and 9 at 5 min. He required supplemental oxygen: initially by continuous positive airway pressure (CPAB); later, by nasal canula. A patent ductus arteriosus (PDA) was detected by echocardiogram; and closure obtained with Indocin. Hyperbilirubinemia resolved with phototherapy. Parenteral nutrition was weaned to breast milk (10 ml q 3 hours).

**Fig. 1:**
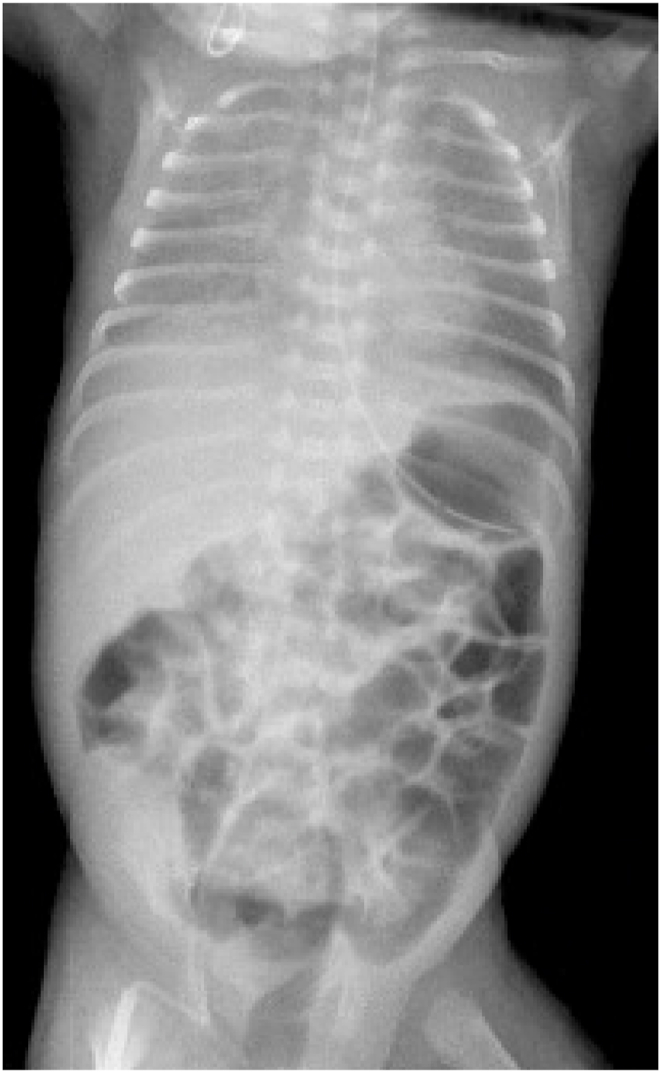
The initial X-ray shows ileus, which causes elevated diaphragms and atelectasis, (the ground glass appearance of the lungs).

**Fig. 2:**
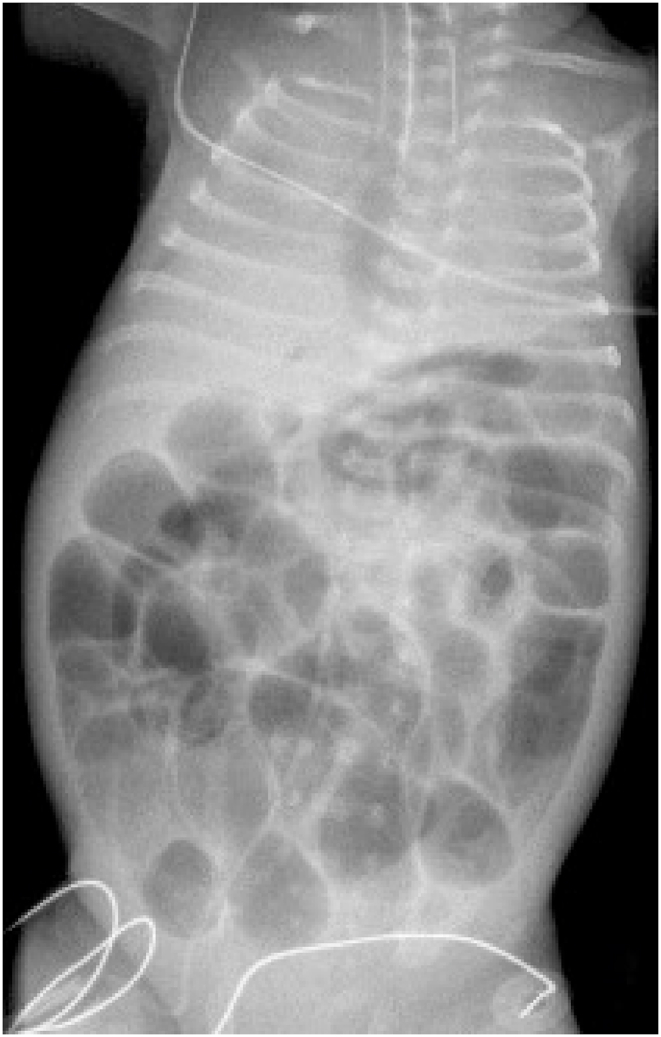
Post-intubation, increased abdominal distension.

**Fig. 3:**
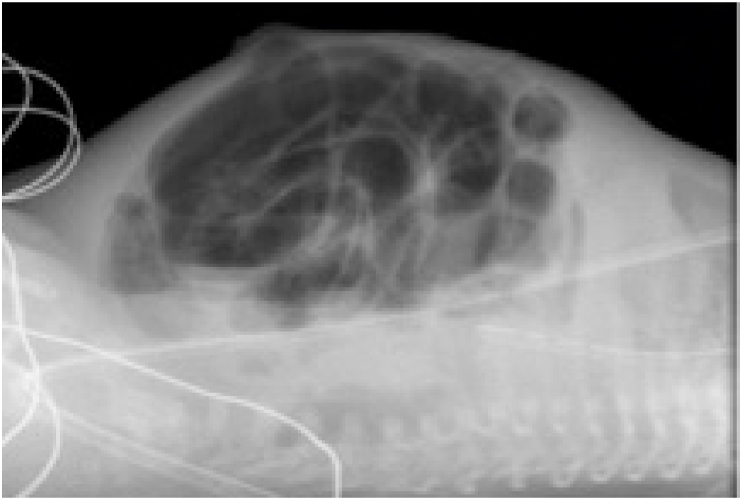
No pneumatosis or pneumoperitoneum is present.

At three-weeks- age, he developed apnea/bradycardia episodes. Radiographs showed haziness of the lungs and ileus (Fig. 1, Fig. 2). Antibiotics were begun and feedings were discontinued. He was intubated (Fig. 3), but he continued to deteriorate; and ultimately, he became hypotensive and oliguric. Dopamine and epinephrine were infused. His WBC and platelet count plummeted, and laboratory data showed mixed acidosis with hyperkalemia, hyperglycemia, and hyponatremia.

**Fig. 4:**
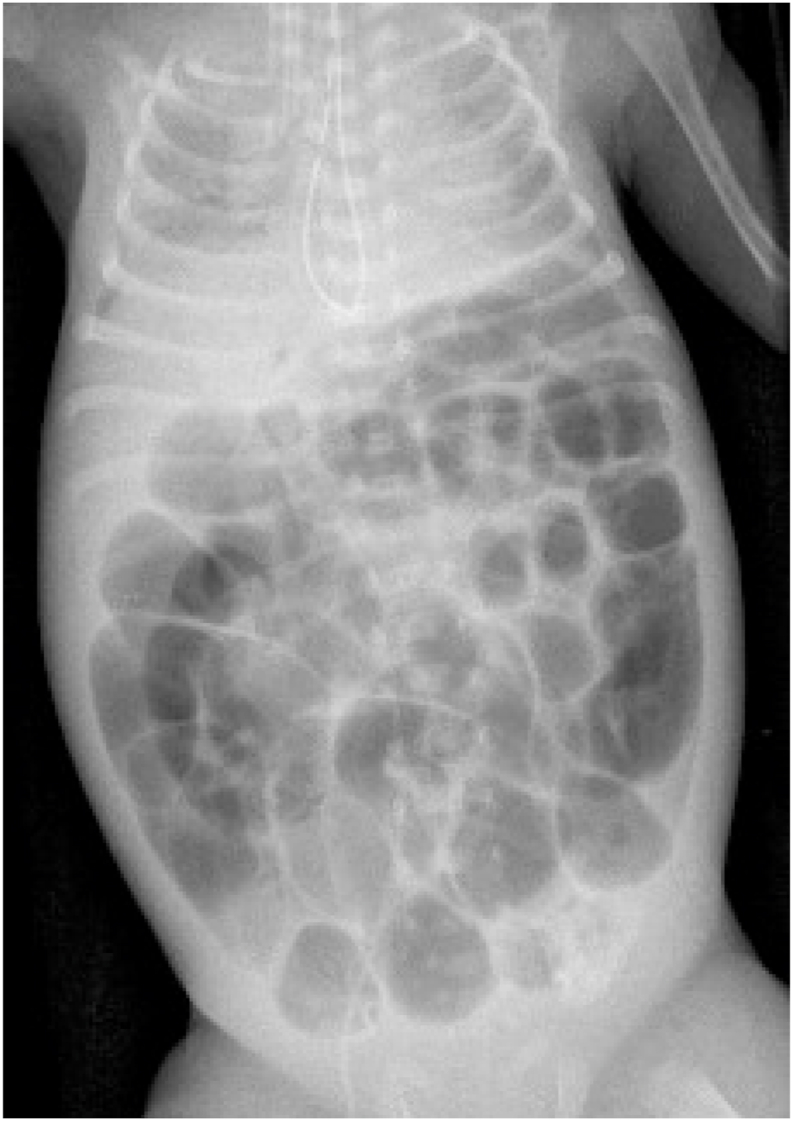
The gastroesophageal junction is compressed by the intra-abdominal pressure, obstructing passage of the orogastric tube into the abdomen. Note the orogastric tube's “U turn” above the diaphragm.

Pediatric surgery consultation was called because the neonatologist was unable to pass an orogastric tube to decompress the baby's massively distended abdomen (Fig. 4).

ABG prior to laparotomy: pH 6.75, pCO2 137, pO2 58, and HCO3 18, and base deficit −20.

ABG after decompressive laparotomy: pH 7.07, pCO2 31, pO2 308, HCO3 8, and base deficit −20.

Upon opening the baby's abdomen, the entire intestine and even the liver appeared ischemic. Decompression restored blood flow to the liver and foregut; but unfortunately, the intestinal necrosis.

### 2.1 Necrotizing enterocolitis [3]

**Fig. 5:**
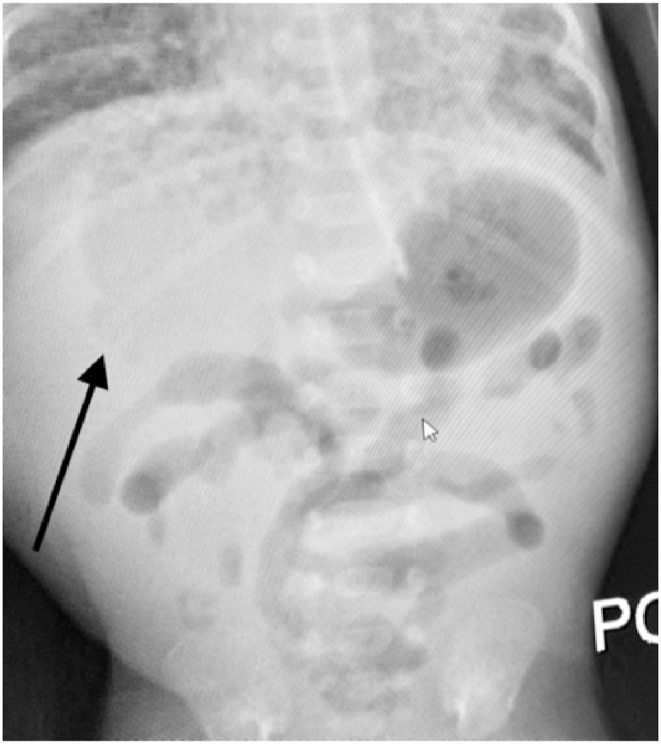
Pneumoperitoneum (football sign).

**Fig. 6:**
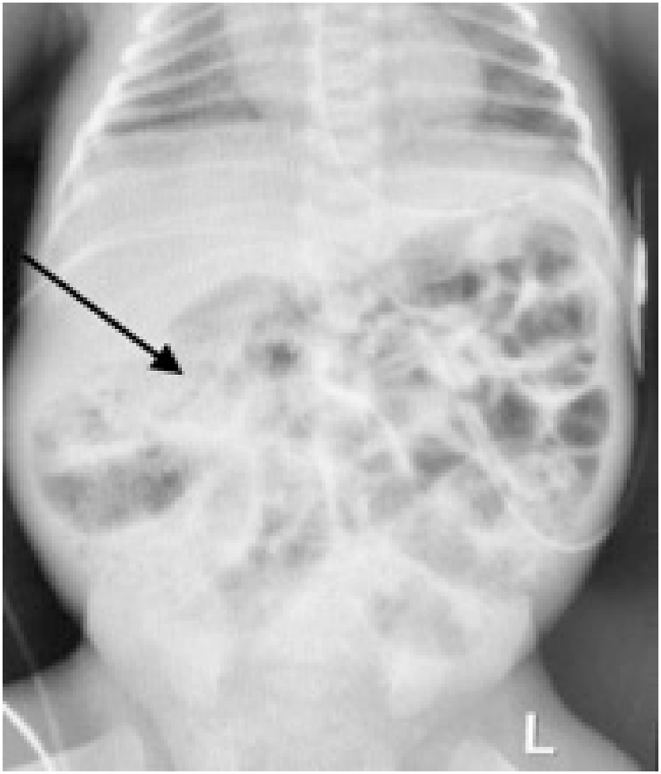
Pneumatosis (arrow).

**Fig. 7:**
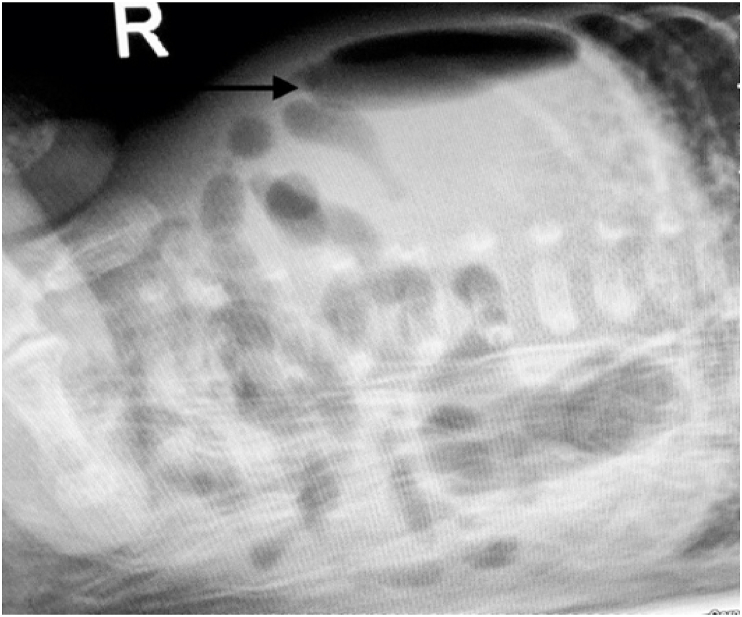
Pneumoperitoneum (note the air above the liver).

Despite reams of research, insights, and innovations, necrotizing enterocolitis (NEC) remains a dreaded perinatal complication. Suddenly a small premature baby develops signs of sepsis: lethargy, apnea/bradycardia, feeding intolerance, and abdominal distension. The clinician's dual task is to treat the sepsis and determine its source. The intestinal gas pattern may be nonspecific, an ileus pattern; or it may be diagnostic, if pneumatosis is present Fig. 6). Pneumoperitoneum is an unequivocal indication for surgical exploration. It may denote either NEC or spontaneous intestinal perforation (Fig. 5, Fig. 7).

### 2.2 Pathophysiology

Stress triggers a “diving reflex”, whereby blood is shunted away from the splanchnic circulation to the brain, heart, lungs, and kidneys. Ischemia makes the intestine more susceptible to bacterial invasion, especially if the anti-microbial and anti-inflammatory factors in breast milk are not provided. Diminished gastric and pancreatic secretions, and ineffective peristalsis favor bacterial overgrowth. An immature immune system is less able to recognize and destroy pathogens. Immature goblet cells are less able to produce mucin which protects the mucosal “tight junctions”. Dysregulation of mucosal blood flow and perturbations in the inflammatory cascade allow release of cytokines (PAF, IL) causing vasoconstriction and increased mucosal and endothelial permeability. Recruitment of cytotoxic granulocytes and other inflammatory agents inhibit reparation of injury.

**The clinical setting of necrotizing enterocolitis is fairly consistent.** Despite an occasional outlier - an unstressed, term baby fed breast milk - NEC is predominantly a disease of premature infants. The following measures are employed prophylactically. Antacids are not given routinely, because diminishing gastric acidity encourages bacterial colonization of the upper GI tract (and ventilator associate pneumonia). Antibiotics are utilized more judiciously. Mothers are encouraged to nurse their babies; and pooled breast milk is utilized, if available.

**Fig. 8:**
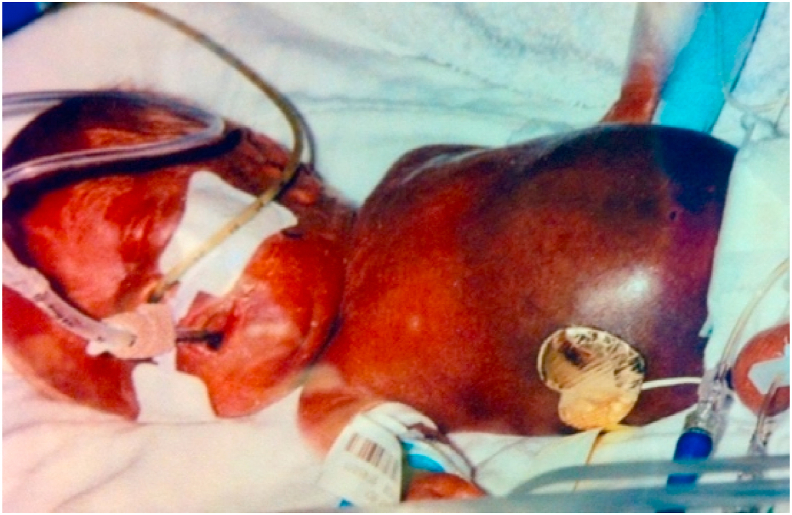
Abdominal wall discoloration from leakage of bile.

**Fig. 9:**
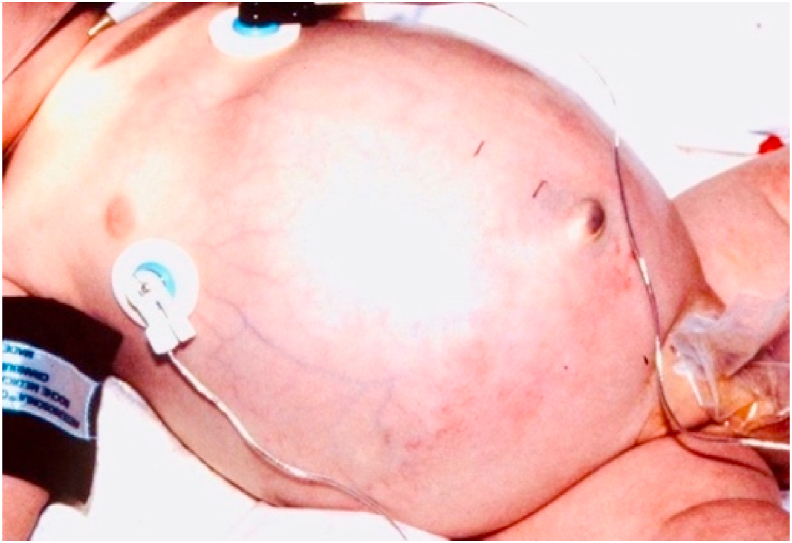
Abdominal erythema denoting peritonitis from intestinal inflammation or necrosis.

Juxtaposed to this consistency is its **astonishingly variable clinical presentation.** NEC may occur suddenly in an unstressed “feeder and grower”, or a in baby who is struggling with an unrelated illness. An intestinal perforation may present with sudden distension from pneumoperitoneum (Fig. 5, Fig. 7) or with only discoloration of the abdominal wall from leakage of bile (Fig. 8). If the perforation is walled-off, the presentation may simulate intestinal obstruction. Abdominal tenderness and erythema may indicate an underlying abscess or intestinal necrosis (Fig. 9). The presentation may be entirely nonspecific: desaturation episodes, feeding intolerance and hematochezia, or abdominal distension and generalized tenderness.

**The operative findings are equally disparate.** The terminal ileum is involved most frequently since goblet cells are scarce and Peyer's patches numerous, resulting in less mucin and more lymphocytes. Or the terminal ileum may be spared, and the jejunum hit or the colon, with segmental or total involvement. Diseased intestine may be interspersed with segments of healthy bowel. Sometimes a watershed line, indicating where perfusion ceased, snakes along the mesentery. The area proximal to this line is viable; distally, there is mesenteric thrombosis, and the adjoining bowel is infarcted. Occasionally everything - stomach, small intestine, and colon, even the rectum - is nonviable. The latter two configurations are characteristic of the abdominal compartment syndrome.

**Babies who develop the abdominal compartment syndrome** are typically “feeders and growers”.

They suddenly decompensate and require ventilatory assistance and ultimately intubation. Initially, the abdomen is distended but soft. Abdominal radiographs show an ileus pattern, uniform distension of the intestine; this may be attributed to assisted ventilation or sepsis, especially since neither pneumatosis nor pneumoperitoneum is identified (Fig. 5, Fig. 6, Fig. 7).

This clinical presentation is ominous because the disease progresses so rapidly. The baby's abdomen becomes tensely distended and rigid. Ventilation is increasingly difficult. Ultimately, the baby develops profound shock. Radiographs show increasing distension with thinning of the bowel wall (Fig. 3, Fig. 4). Urgent laparotomy is indicated; but it may be too late. There may be *pan necrosis* of the intestine.

### 2.3 **Abdominal Compartment Syndrome** [[4], [5], [6]]

Increasing pressure within a closed anatomic space adversely affects perfusion and the viability of the organs within that space. Intra-abdominal pressure is assessed by measuring the pressure within a hollow viscus such as stomach or bladder. In adults, the normal intra-abdominal pressure is 5 mm Hg. It fluctuates with respiration, activity, and body mass index. The abdominal perfusion pressure (APP) is the mean arterial pressure (MAP) minus the intra-abdominal pressure (IAP). In normal adults, the APP is < 50 mm Hg.

The abdominal compartment syndrome (ACS) is considered primary, when the etiology is a traumatic injury causing hemorrhagic shock. It is deemed secondary in the absence of intra-abdominal pathology, as in burn injury or sepsis. A common factor causing ACS is massive fluid resuscitation utilizing crystalloids.

**Increased intra-abdominal pressure compromises cardiopulmonary function and perfusion of the intra-abdominal viscera.** Cardiac filling pressure is deceptively elevated. Venous return (preload) is decreased; there is pooling of blood in the splanchnic and lower extremity vascular beds, and cardiac output falls. Cardiac work is increased; the elevated intra-abdominal pressure resists aortic outflow, and decreased MAP stimulates sympathetic neural discharge and release of renin, aldosterone and ADH, which increases peripheral vascular resistance (afterload). Oliguria occurs because of decreased MAP, renovascular compression, and direct pressure upon the kidneys. Chest wall edema decreases the compliance of the thoracic cavity; and elevation of the diaphragm causes hypoventilation and hypercarbia, which necessitates utilization of airway pressures that exceed the increased the intra-thoracic pressure. Compression and edema of the pulmonary parenchyma cause ventilation perfusion (V/Q) mismatch and hypoxia. Compression of the thin-walled mesenteric veins causes venous congestion and interstitial swelling and bowel wall edema. Splanchnic hypoperfusion causes cellular hypoxia and mucosal acidosis. Breakdown of the mucosal barrier permits bacterial translocation and sepsis.

**Hypoperfusion (shock) causes refractory metabolic acidosis**. The ischemic liver cannot metabolize lactate. Reperfusion injury activates inflammatory cells releasing cytokines. Multisystem organ failure (systemic inflammatory syndrome) and death ensue.

**The ACS in premature infants is caused by the accumulation of intestinal air rather than interstitial edema.** Insertion of an orogastric tube is useless because the stomach is empty. Passage of an orogastric tube through the gastroesophageal junction may be impossible because of the elevated intra-abdominal pressure (Fig. 4).

## 3 Conclusion

This case report highlights a frequently overlooked complication of NEC, the abdominal compartment syndrome. Remarkably, surgical consultation was sought not to operate and decompress the abdomen, but to pass an orogastric tube. Neonatology was oblivious to the cause of their difficulty (and the baby's demise). Their focus was upon treating the infection and its consequences: septic shock and multisystem organ failure.

This case is illustrative of an error that besets all clinicians: a sign of disease is perceived but its significance is misinterpreted or overlooked. A septic baby develops abdominal distension that compromises ventilation. Gastric distension may be relieved by an orogastric tube but not dilatation of the intestines; moreover, the intra-abdominal pressure may be so great as to collapse the gastroesophageal junction, making passage of the tube impossible.

The significance of abdominal distension is missed entirely; it is not peripheral to the baby's disease process; it is a major component, and an urgent laparotomy is needed.

Our plea is simply, don't overlook another pathogenic process, the abdominal compartment syndrome. Laparotomy is too late, when it reveals a watershed line, snaking along the mesenteric intestinal junction, indicating where perfusion ceased, and necrosis began.

Consent for the original case report was obtained, but that was >10 years ago. I have rewritten the case report because I believe it is instructive.

I am the sole author, and I meet the current ICMJE criteria.

I have no competing financial interests or personal relationships that influenced this report.

Fig. 8 is one of the photographs at the NICU entrance, Saint Francis Hospital, Wichita KS.

Fig. 9 is from a teaching file, Le Bonheur Children's Hospital, Memphis TN.

The other photographs were taken by the author and are used by permission.

## Provenance and peer review

Not commissioned, externally performed.

## Sources of funding

None.

## Ethical approval

This report does not contain any personal information that could lead to the identification of this baby. The figures (photographs) are from my personal collection.

## Consent

This report does not contain any personal information that could lead to the identification of this baby. The figures are from my personal collection.

## Author contribution

I am the sole author.

## Registration of research studies NA

1. Name of the registry: NA
2. Unique Identifying number or registration ID: NA
3. Hyperlink to your specific registration (must be publicly accessible and will be checked): NA

## Guarantor

I am the sole guarantor.

## Declaration of competing interest

The author declares that he has no known competing financial interests or personal relationships that could have appeared to influence the work reported in this paper.
